# Nuclear Transit and HIV LTR Binding of NF-κB Subunits Held by IκB Proteins: Implications for HIV-1 Activation

**DOI:** 10.3390/v11121162

**Published:** 2019-12-16

**Authors:** Sohrab Z. Khan, Sofia Gasperino, Steven L. Zeichner

**Affiliations:** 1 Department of Pediatrics, Child Health Research Center, and the Pendleton Pediatric Infectious Disease Laboratory, University of Virginia, Charlottesville, VA 22908, USA; sohrabzafar@gmail.com (S.Z.K.); SLG7S@hscmail.mcc.virginia.edu (S.G.); 2 Department of Microbiology, Immunology, and Cancer Biology, University of Virginia, Charlottesville, VA 22908, USA

**Keywords:** HIV-1, latency, activation, reservoir, IκB, IκBα, IκBε, NF-κB

## Abstract

No effective therapy to eliminate the HIV latently infected cell reservoir has been developed. One approach, “shock and kill”, employs agents that activate HIV, subsequently killing the activated infected cells and/or virus. Shock and kill requires agents that safely and effectively activate HIV. One class of activation agents works through classical NF-κB pathways, but global NF-κB activators are non-specific and toxic. There exist two major IκBs: IκBα, and IκBε, which hold activating NF-κB subunits in the cytoplasm, releasing them for nuclear transit upon cell stimulation. IκBα was considered the main IκB responsible for gene expression regulation, including HIV activation. IκBε is expressed in cells constituting much of the latent HIV reservoir, and IκBε knockout mice have a minimal phenotype, suggesting that IκBε could be a valuable target for HIV activation and reservoir depletion. We previously showed that targeting IκBε yields substantial increases in HIV expression. Here, we show that IκBε holds c-Rel and p65 activating NF-κB subunits in the cytoplasm, and that targeting IκBε with siRNA produces a strong increase in HIV expression associated with enhanced c-Rel and p65 transit to the nucleus and binding to the HIV LTR of the activating NF-κBs, demonstrating a mechanism through which targeting IκBε increases HIV expression. The findings suggest that it may be helpful to develop HIV activation approaches, acting specifically to target IκBε and its interactions with the NF-κBs.

## 1. Introduction

While combination antiretroviral therapy (cART) can effectively control disease in a patient infected with HIV-1, cART does not cure a patient of the infection, due to the existence of a persistent reservoir of long-lived latently infected cells, largely CD4+ memory T cells (recently reviewed in [1,2,3,4]). Considerable interest has centered on developing ways to attack, deplete, and ideally eliminate the long-lived reservoir of latently infected cells. One possible approach to attacking the latent reservoir has been termed “shock and kill” (reviewed in [5]), in which a patient would be treated with agents that activate latent HIV, then given antiviral or immunologic therapies that would destroy the resulting activated viruses and their host cells.

Much work has been done to develop effective HIV activators or latency-reversing agents (LRAs)—the “shock” component of “shock and kill.” Shock and kill strategies are theoretically appealing, but unfortunately have generally proved ineffective in clinical settings, and in some cases have been shown to be highly toxic. Available LRAs also lack cell specificity and their broad mechanism of action yields toxicity, off-target effects, and limited dosing range [6]. While blocks to HIV activation occur at several different levels, transcriptional initiation is one key level; and adequate transcriptional initiation must be present for other levels, such as transcriptional elongation and splicing [7] and stochastic fluctuations [8], to come into play.

LRAs can be classified according to their mechanisms of action, for example LRAs that act epigenetically, and T-cell activators [6]. Epigenetic activators studied have included histone deacetylase (HDAC) inhibitors [9,10,11,12,13], DNA methylation inhibitors [14], and bromodomain/extraterminal domain (BET) inhibitors [15]. T cell activator LRAs include agents that act through conventional T cell activation pathways [16], such as IL-2 and the OKT3 monoclonal antibody (mAb) against CD3 [17]; diacyl glycerol analog protein kinase C (PKC) agonists, such as phorbol esters (e.g., phorbol 12-myristate 13-acetate, TPA, or PMA, reviewed in [18,19]); less toxic cell activators like bryostatin-1 [20]; and potentially mTOR [21] and JAK inhibitors [6,22]. Most T cell activators act through the NF-κB pathway, releasing activating NF-κB subunits from IkBα for transit to the nucleus, with subsequent increases in HIV transcriptional initiation [19]. HIV activation strategies employing cytokines and chemokines, working through NF-κB, have long been studied [17,23,24,25,26]. However, such agents have toxicities that make them clinically unacceptable or were shown to be ineffective against the latent reservoir in vivo, or both. Small molecules have also been used to activate HIV via NF-κB-related pathways. The best known of these is the diacylglycerol mimetic phorbol myristyl actetate (PMA, TPA) [27] and its derivatives [28,29,30], but phorbol esters are oncogenic and induce reactive oxygen species targets. Even the less toxic derivatives still show significant toxicity and a poor ability to target the latent reservoir [18,19,28]. Agents mechanistically related to known LRAs that have specificity for latent reservoir cells or specificity for HIV activation, compared to non-HIV activation targets, could serve as more effective and less toxic LRAs, useful alone or in combination with other HIV activators.

For expression, the HIV promoter, the long terminal repeat (LTR), requires basal cellular transcription factors, plus inducible factors, notably NF-κB family members, and other host cell factors [27,31,32,33,34,35,36,37,38]. Other cellular activation-dependent, cell-type dependent, or differentiation-dependent factors also contribute to LTR activity [39,40,41,42,43,44,45]. NF-κB has long been known as a key gene expression regulator for many cells [46,47,48,49]. Five different related factors comprise the NF-κB family: p50, p53, p65 (RelA), c-Rel, and RelB. The proteins share an N-terminal Rel-homology (RHD) domain involved in DNA binding and homo- and hetero-dimerization. NF-κBs bind as dimers to their binding sites in promoters. Some NF-κBs, when bound to promoters, recruit co-activators and co-repressors, including HIV LTR activators. Three NF-κBs: p65, c-Rel, and RelB, have C-terminal transcription activation domains (TAD) which mediate interactions with activators. Homodimers of p50 or p52, which lack TADs, inhibit transcription. In unactivated HIV LTRs, NF-κB p50 homodimers bind LTR NF-κB sites, inhibiting expression, a phenomenon that also involves recruitment of HDACs to the LTR [50]. Upon stimulation, inhibitory p50 homodimers are displaced by TAD-containing NF-κBs (e.g., p50/p65, p50/c-Rel heterodimers), activating expression [51,52]. Different TAD-containing NF-κBs can partially compensate for each other if one or the other has been knocked out or mutated, but the different TAD-containing NF-κB subunits have distinct specificities and, presumably, functions within the cell [53,54].

In the absence of stimulation, NF-κB proteins are held in the cytoplasm, complexed as inactive forms with IκBs. The IκB family includes three typical IκB proteins, IκBα, IκBβ, and IκBε; their precursor proteins, p100 and p105; and two atypical IκB proteins, Bcl-3 and IκBζ [49]. The two atypical IκB proteins, Bcl-3 and IκBζ, have less clearly understood functions [55,56,57,58]. The TNFα pathway for NF-κB-dependent activation offers a classical example of transcriptional activation via NF-κB [48,49]. In a simplified account, TNFα binds to its receptor, TNF-R, which recruits adaptor proteins (TRAFs and RIP) that interact with TNF-R cytoplasmic domains. TRAFs and RIP recruit an IKK complex, which includes the NEMO scaffold protein and α and β catalytic IKK subunits, to activate the complex. Activated IKK phosphorylates an IκB, initiating ubiquitination and proteamsome degradation, releasing NF-κBs that transit to the nucleus and activates gene expression.

The IκBs have different functions and activities. IκBα is the best understood. Most cytoplasmic p65/p50 heterodimers bind IκBα. NF-κB can activate IκBα expression, creating a negative feedback loop [59,60]. IκBα knockout (KO) mice have a severe phenotype [61], dying at 7–10 days of age. IκBα-deficient fibroblasts respond to TNFα and maintain NF-κB in the cytoplasm prior to stimulation, suggesting that other IκBs compensate, but NF-κB nuclear localization is longer. IκBβ’s function is less clear. The IκBβ promoter does not respond to cell stimulation. IκBβ binds p65 and C-rel more, and binds p50 less [62,63]. IκBε’s physiologic function is even less well understood [64,65]. IκBε is expressed mainly in T cells of the thymus, spleen, and lymph nodes [66], which coincide with the locations of key reservoirs of latent HIV. IκBε KO mice have a relatively normal phenotype vs. the lethal phenotype of IκBα KO mice [66]. IκBε KO mice are identical to wildtype mice in appearance and histology, and breed normally. The main differences between them are decreased CD44-CD25+ T cells, and increased production of IL-1α, IL-1β, IL-1Rα, and IL-6 mRNAs in macrophages. Since IκBε KO mice have a minimal phenotype, pharmacologic targeting of IκBε is expected to be relatively safe.

We previously conducted a systematic small interfering RNA (siRNA) knockdown study targeting the major IkBs (IkBα, IkBβ, and IkBε), to investigate how the different IkBs might help mediate HIV-1 latency [67]. We found that knocking down IkBβ did not activate HIV-1, and knocking down IkBα activated HIV-1, as expected. We unexpectedly found that knocking down IkBε was much more effective at activating HIV-1 than knocking down IkBα. Since IkBε is highly expressed in cells and tissues that harbor a large fraction of the HIV latent reservoir [66], and since IkBε knockout (KO) mice have a minimal phenotype [59,66], HIV activation strategies specifically targeting IkBε could plausibly play a helpful role in activating HIV in “shock and kill” HIV reservoir attack strategies.

If targeting IkBε were to be explored as a more specific HIV activation strategy, it would be helpful to have a better understanding of the mechanisms responsible for the effective activation of HIV by IkBε. LRAs specifically targeting the IkB-NF-κB pathway, like prostratin and bryostatin, are directed to the IkBα-NF-κB pathway with understandable consequences for enhanced toxicity and a lack of specificity. Here, we show that when we knock down IkBε, we observe, as before, potent HIV activation [67]. We also show that the activation due to IkBε knockdown is associated with the transit to the nucleus of activating c-Rel and p65 subunits, and increased binding of these NF-κBs to the HIV LTR. These findings, along with the more prolonged knockdown kinetics of IkBε that we previously observed [67], and the knowledge that IkBε is abundantly expressed in cell types that constitute the latent reservoir and the minimal phenotype of IkBε knockout mice, lends additional support to the possibility of specifically targeting IkBε to activate HIV-1 in the context of a “shock and kill” HIV reservoir depletion strategy.

## 2. Materials and Methods

### 2.1. Cell Lines and Transfection

We utilized a latently infected promonocytic cell line, U1 [68] derived from chronically infected U937, and lymphocytic cell lines J1.1 and ACH-2 cells [69,70,71]. The cells were seeded 24 h prior to transfection at a cell concentration of 2 × 10^5^ cells/mL in RPMI (Life Technologies, Carlsbad, CA, USA) with 1% l-glutamine (Life Technologies), 10% fetal bovine serum (FBS) (Hyclone), 100 IU/mL penicillin, and 100 µg/mL streptomycin (Sigma, St. Louis, MO, USA) in 5% CO_2_ at 37 °C. On the day of transfection, specific siRNAs at the desired concentrations were mixed with 2 million U1 cells, resuspended in Nucleofector solution (Lonza, Alpharetta, GA, USA), and transfected using Nucleofactor 2D. Once transfected, cells were transferred to 60 mm dishes that contained 3 mL of pre-warmed 1 phosphate-buffered saline (PBS). Plates were incubated for 3 h in 5% CO_2_ at 37 °C. Cells were transferred to a 5 mL tube and centrifuged at 1200 rpm for 5 min. The cell pellets were resuspended in 7 mL RPMI supplemented with 10% FBS, and incubated in a 60 mm dish for 36 h at 37 °C. Twenty four hours post transfection, 750 µL culture was taken to extract total RNA using an RNeasy Mini Kit (Qiagen, Germantown, MD, USA). Thirty six hours post transfection, cells were pelleted, and supernatants were stored at −80 °C for further use. Knockdown efficiencies observed in these studies were comparable in the data presented here, and in additional and previously published experiments [67].

### 2.2. Nuclear and Cytoplasmic Extract Preparation

A total of 5–8 × 10^6^ U1 cells were harvested 36 h after siRNA transfection and washed with ice cold 1× PBS. Nuclear and Cytoplasmic extracts were prepared using NE-PER^TM^ Nuclear and Cytoplasmic Extraction Reagents (Thermo Fisher Scientific, Rockford, IL, USA) according to the manufacturer’s instructions. The protein extracts were estimated using Bradford reagent (BioRad, Hercules, CA, USA) according to the manufacturer’s instructions and equal amounts (15–20 µg) of nuclear and cytoplasmic extracts were used for immunoblot.

### 2.3. Immunoblotting

For protein isolation, the cell pellet was lysed in TN-lysis buffer (20 mM Tris-Cl, 150 mM NaCl, 1 mM EDTA, 0.5 mM PMSF, 0.5% NP-40, and 1× protease inhibitor) by incubating for 45 min on ice with intermittent agitation. The samples were centrifuged at 13,000 rpm for 15 min at 4 °C and clarified lysates were transferred to the fresh tubes. The protein present in the clarified lysate was estimated using a Bradford assay (BioRad), according to the manufacturer’s instructions. Twenty five micrograms of the total clarified isolated protein was mixed with a 4× Laemmli buffer, heated at 95 °C for 10 min, and loaded on a NuPAGE 4–12% Bis-Tris pre-cast gel (Life technologies). Proteins were transferred onto a nitrocellulose membrane (0.45 μm, BioRad) using 1× NuPAGE Transfer Buffer (Invitrogen Life Technologies, Carlsbad, CA, USA). The blots were blocked with 5% non-fat milk for 1 h prior to immunoblotting with anti-GAPDH (Abcam), anti-Lamin A/C (Peirce), anti-p65 (Abcam), anti-c-Rel (Abcam), and anti-HIV Gag-p24 (AIDS Reagent Program). Proteins were detected with goat anti-mouse IgG-HRP (Invitrogen), goat anti-rabbit IgG-HRP, and chemiluminescent substrate (Thermo Scientific). Immunoblots were imaged using a ChemiDoc MP instrument (BioRad). Composite figures were produced in Affinity Photo and Affinity Designer.

### 2.4. Chromatin Immunoprecipitation Assay (ChIP)

Chromatin immunoprecipitation (ChIP) assays were performed using ChIP-IT Express Enzymatic Kit (ActiveMotif, Carlsbad, CA, USA) according to the manufacturer’s instructions. Briefly, 5–8 × 10^6^ U1 cells were fixed 36 h post transfection in 1% formaldehyde for 15 min at room temperature. The fixed cells were then washed using wash buffer 1 (0.25% Triton X-100, 10 mM EDTA, 0.5 mM ethylene glycol bis(β-aminoethyl ether) N,N′-tetraacetic acid, 10 mM HEPES (pH 7.5), 1 mM PMSF, 10 mM sodium butyrate, and 2× protease inhibitor cocktail) and wash buffer 2 (0.2 M NaCl, 1 mM EDTA, 0.5 mM ethylene glycol bis(β-aminoethyl ether) N,N′- tetraacetic acid, 10 mM HEPES (pH 7.5), 1 mM PMSF, 10 mM sodium butyrate, and 2× protease inhibitor cocktail) and lysed in lysis buffer (1% SDS, 10 mM EDTA, and 50 mM Tris-HCl (pH 8.1)). Sonication cycles (10 × 10 s with 10 s hold on ice between each pulse) were then performed to shear chromatin using a Bioruptor (ActiveMotif). The lysate was precleared with protein A/G beads (ActiveMotif); and the precleared lysate was immunoprecipitated with mouse antihuman RNA Pol II antibody, or with the negative control IgG (ActiveMotif). The immune complexes were then pulled down by protein A/G beads and washed thrice with RIPA wash buffer (0.1% SDS, 1% sodium deoxycholate, 150 mM NaCl, 10 mM sodium phosphate (pH 7.2), 2 mM EDTA, and 1% IGEPAL) and then thrice with TE (10 mM Tris (pH 8.0) and 1 mM EDTA). Elution of the complexes were then performed in 0.1 M NaHCO_3_, 0.1% SDS, and 10 mM DTT. DNA was extracted after reverse cross-linking, proteinase K treatment, and phenol chloroform extraction. The eluted DNA was dissolved in 50 µL of 1× TE. ChIP-PCR was performed in a 25 µL reaction mixture containing 1× Real-time PCR master mix (Promega, Madison, WI, USA) and 10 pmol of LTR primers (ChIP2_LTR_f: 5′-CCGAGAGCTGCATCCGGAGT-3′, ChIP2_LTR_r: 5′-ACTGCTAGAGATTTTCCACACT-3′).

### 2.5. HIV-1 RNA Quantitation

Total RNA was extracted from the cell pellets 24 h after siRNA transfection, using an RNeasy Mini Kit (Qiagen) according to the manufacturer’s instructions. The extracted RNA was quantified using a NanoDrop HD1000 Spectrophotometer (Thermo Scientific). The purity of the RNA samples was estimated based on the 260:280 absorbance ratio, and samples were required to have ratios ≥2. 500–750 µg of total RNA per sample was used for reverse transcription reactions using iScript^TM^ Reverse Transcription Supermix for qRT-PCR (Bio-Rad) according to the manufacturer’s protocol. Briefly, the RNA was mixed with 4 μL of 5× iScript RT Supermix, and RNase-free water (Qiagen) was added to the reaction to make up a volume of 20 μL. For negative control or no reverse transcriptase control (NRTC) reactions, we used a reaction mixture made by mixing RNA, water, and 4 μL of 5× iScript no-RT Supermix. The reactions were incubated for 5 min at 25 °C for primer annealing, 60 min at 42 °C for reverse transcription, and then at 85 °C for 5 min for enzyme inactivation in a Thermal Cycler (Bio-Rad). The cDNA reactions were diluted tenfold, and 2 μL of diluted cDNA was used in real time PCR reactions. For the qPCR reactions, we used the TaqMan master mix system (Applied Biosystems, Foster City, CA, USA), TaqMan probes specific for HIV-1 late RNA (unspliced RNA) (IDT), and human GAPDH (Applied Biosystems). The sequences of primer sets used to amplify HIV-1 unspliced RNA were 5′ -ATAATCCACCTATCCCAGTAGGA GAAAT-3′ and 5′TTTGGTCCTGTGCTT ATGTCCAGAATGC [72]. A FAM-TAMRA-labeled probe, 5′ -ATCCTGGGATTCAATAAAATAGTAGAGATGTATAGCCCTAC- 3′, was used for quantitation of late viral RNA species [67]. The thermal cycling conditions were 50 °C for 2 min, and an initial denaturation at 95 °C for 15 s, followed by 40 cycles at 95 °C for 15 s, and 60 °C for 60 s using the Applied Biosystems 7500 Fast Real Time PCR detection system. All reactions were performed in 20 μL final volume, with human GAPDH used as endogenous control, and NRTC as negative control. The amount of PCR product was determined by the comparative 2^−ΔΔCt^ method [73], with each sample normalized to human GAPDH and expressed as a fold-increase versus untreated controls.

### 2.6. Image Quantitation

Quantitative data was extracted from gel images using ImageJ version 2.0.0-rc-69/1.52p, using the FIJI interface, with included plugins.

### 2.7. Statistical Analyses

Statistical analyses were conducted using log-transformed values, 2-tailed Student’s *t*-test, 2-sample unequal variance, using Excel and R (version 3.6.1) with the RStudio user interface and the tidyverse package. Experiments were repeated at least three times, with representative gels shown. Some graphs were also generated with Graphpad. Error bars shown, indicate standard deviations.

## 3. Results

To explore the mechanisms underlying the large increases in HIV expression seen particularly when IκBε is knocked down, we confirmed that IκBε siRNA knocks down IκBε expression at the RNA and protein levels; showed that IκBε knockdown is associated with HIV activation; and determined cytoplasmic and nuclear localization of two activating, canonical examples of TAD-containing NF-κB subunits from the Rel and NF-B families, c-Rel and p65. c-Rel and p65 are activated via inflammatory and pathogenic signals that are thought to be likely mediators of HIV activation [74].

### 3.1. siRNA Transfection and HIV Transcription Activation

**Figure 1:**
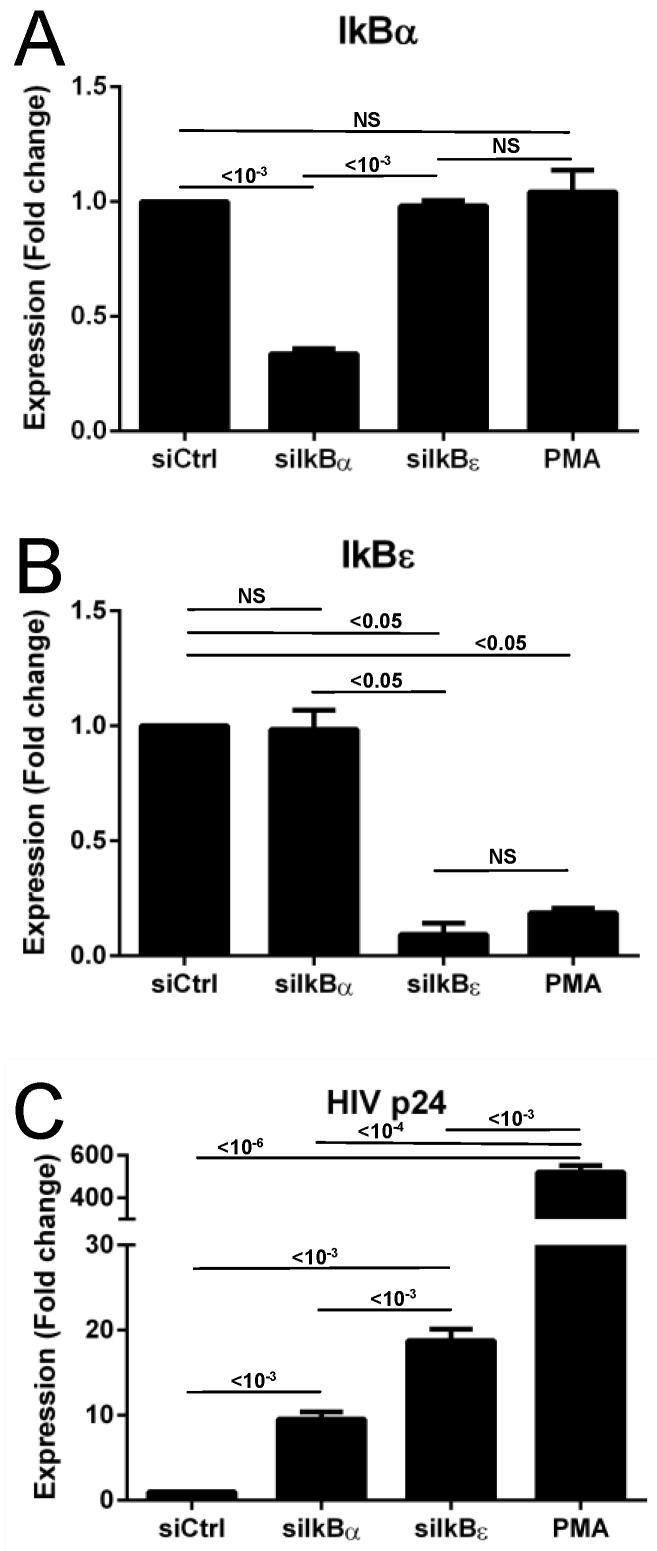
Effects of IκB siRNAs on IκB RNA and HIV p24 RNA expression in U1 cells. (**A**) Effects of IκBα, IκBε, and control siRNA, and positive control activator PMA on IκBα RNA expression. (**B**) Effects of IκBα, IκBε, and control siRNA, and positive control activator PMA on IκBε RNA expression. (**C**) Effects of IκBα, IκBε, and control siRNA, and positive control activator PMA on HIV p24 RNA expression. *P*-values, per two-tailed Student *t*-test performed on log-transformed values with unequal variance, are indicated on the graphs.

To determine the effects of IκBα and IκBε siRNA on IκBα, IκBε, and HIV expression at the RNA level, we transfected the different IκB siRNAs into U1 cells latently infected with HIV in parallel, along with a control siRNA, while also treating cells in parallel with PMA as a positive control. We isolated RNA and assayed for IκBα, IκBε, and HIV p24 RNA using a qPCR assay (Figure 1). We found that IκBα siRNA transfection was associated with a decrease in IκBα RNA; minimal decrease in IκBε RNA and IκBε siRNA transfection was associated with a strong decrease in IκBε RNA; and minimal decrease in IκBα. The results also indicated good specificity and minimal crosstalk between the effects of the IκBα and IκBε siRNAs. Interestingly, PMA treatment was associated with decreased IκBε RNA, but not with decreased IκBα RNA, reflecting the feedback loops involved in the NF-κB-dependent pathways regulating the IκBε expression, and providing additional rationale for the strong increases in HIV expression observed with PMA treatment. IκBα siRNA increased HIV expression, but IκBε siRNA increased HIV expression almost twice as much. PMA is well-established as an agent that dramatically increases HIV expression [17,23,24,25,26,27].

### 3.2. IκBα and IκBε siRNA Transfection and IκBα and IκBε and HIV Protein Expression

**Figure 2:**
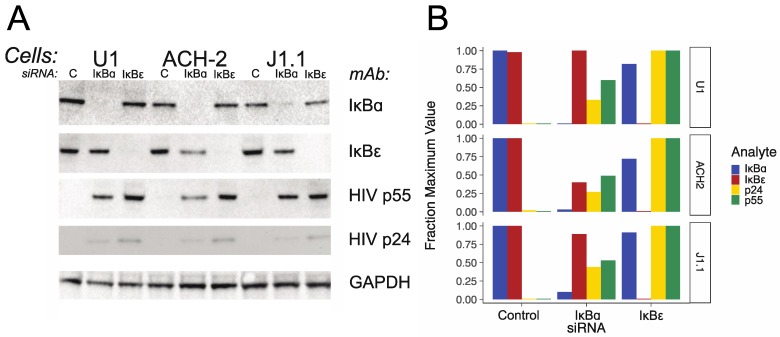
Effects of IκB siRNAs on IκB protein and HIV protein expression in U1, ACH-2, and J1.1 cells. (**A**) Composite immunoblot. Cell lines used in this experiment are indicated at the top of the figure, with the individual siRNA used in the experiments run in each lane indicated beneath. The designations to the right of the figure indicate the target of the mAb used for each immunoblot. (**B**) Immunoblot quantitation. Bands from panel A were quantitated using ImageJ normalized to the GAPDH control, and then for each condition, normalized against the condition yielding the maximum value. The fraction of the maximum values for each condition are indicated on the left side of the panel, cell lines are indicated on the right side of the panel, and the control and/or siRNAs transfected in each experiment are indicated at the bottom of the panel.

To confirm that IκBα and IκBε siRNAs had their expected effects at the protein level for the expression of the IκBs and for HIV protein expression, and was similar in several cell line models of latent HIV infection, we transfected IκBα and IκBε siRNAs into U1, ACH-2, and J1.1 cells; made whole cell protein extracts from the cells; and used immunoblots to test for IκBα and IκBε proteins, HIV p55 and HIV p24, with GAPDH as control. Figure 2A shows a composite of the different immunoblots. Figure 2B shows graphs of the quantitation of the bands, normalized to the GAPDH control, and then normalized to the maximum expression of each band in each cell line transfected with the indicated siRNA. IκBα siRNA decreased IκBα protein in all the cell lines, and IκBε siRNA also decreased IκBε protein in all cell lines. Both IκBα and IκBε siRNA transfection were associated with increases in HIV p55 and p24 proteins, as expected, with transfection with IκBε siRNA again associated with a larger increase in HIV protein expression.

### 3.3. Nuclear Translocation of Activating NF-κB Subunits Associated with Transfection of IκBα and IκBε siRNAs

**Figure 3:**
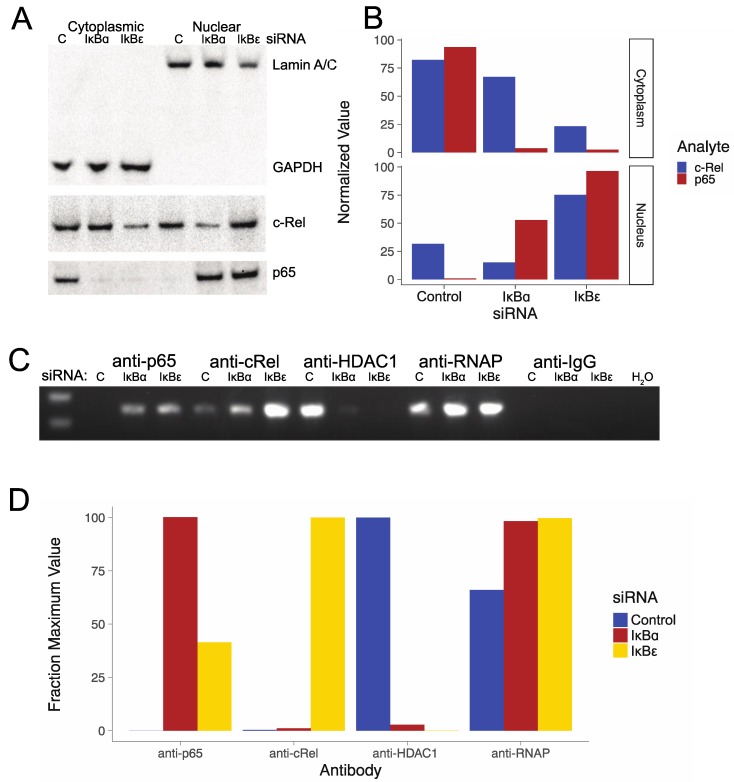
Nuclear transit and chromatin immunoprecipitation assays of NF-κB subnits following IκB knockdown. (**A**) Assays showing transit from cytoplasm to nucleus of NF-κB subunits, c-Rel and p65, following transfection of the cells with IκBα and IκBε siRNAs. The origin of the protein extracts, cytoplasmic or nuclear, is indicated at the top of the figure. The designators on the right side of the figure indicate the mAb use for the immunoblot. (**B**) Quantitation of the bands from panel A—nuclear transit experiments. The c-Rel and p65 bands were normalized to the GAPDH loading control band for the cytoplasmic samples and/or the Lamin A/C loading control band, and then multiplied by an arbitrary constant to expand the range for each set of assays to 100. The normalized values (arbitrary units) are shown on the left, the cytoplasmic or nuclear compartment sources are indicated on the right side of the figure, and the siRNA used in each experiment is indicated at the bottom of the panel. (**C**) HIV LTR chromatin immunopreciptation assays for NF-κB subunits. c-Rel and p65, following transfection of the cells with IκBα and IκBε siRNAs. The top line indicates the primary mAb used in the immunopreciptation, while the indicators on the line beneath indicates the siRNA transfected into the cells to generate the extract assayed in the reactions run in each lane. (**D**) Quantitation for the chromatin immunopreciptation assays. The bands from panel C were scanned and quantitated, and then for each antibody, normalized to the condition of maximum signal set as 100 percent for each antibody, since the ChIP assays performed using each antibody cannot be directly quantitatively compared with each other. Quantitations were not performed for the anti-IgG and H_2_O negative controls because the scanned values for these lanes were essentially zero.

To determine whether knocking down IκBα and IκBε was associated with transit of the activating NF-κB subunits from the cytoplasm to the nucleus (which were required for HIV activation), we transfected IκBα and IκBε siRNAs and a control siRNA into U1 cells and conducted a nuclear/cytoplasmic split, and then evaluated the resulting protein extracts using immunoblots (Figure 3A,B). The results show that nuclear/cytoplasmic split was successful, with the cytoplasmic marker detectable in the protein extracts from the cytoplasmic fractions only, and the nuclear marker Lamin A/C observed in the nuclear fractions only. We found that the c-Rel NF-κB subunit was observed in both the cytoplasmic and nuclear fractions, but that transfection with the IκBε siRNA was associated with greater amounts of c-Rel in the nucleus. We detected only minimal amounts of the p65 NF-κB subunit in the cytoplasm 36h after transfection with IκBα and IκBε siRNAs, with a large increase in the nucleus of the p65 NF-κB subunit after IκBα siRNA transfection, and a larger increase in p65 after transfection with IκBε siRNA.

### 3.4. HIV LTR NF-κB Chromatin Immunoprecipitation After Transfection of IκBα and IκBε siRNAs

To determine whether there were differences in the binding of the activating NF-κB subunits to the HIV LTR after IκBα and IκBε knockdown, we conducted chromatin immunoprecipitation studies following the transfection of IκBα and IκBε siRNAs (Figure 3C,D). We found that transfection of the IκBα and IκBε siRNAs was associated with a large increase in the binding of the activating NF-κB subunits c-Rel and p65, to the HIV LTR, compared to transfection with control siRNA; a large decrease in HDAC1 bound to the LTR, compared to transfection with control siRNA; and a modest increase in the binding of RNAP to the LTR.

## 4. Discussion

Taken together, the results we report in this study and in our earlier study [67] are consistent with the following model: Compared to IκBα, IκBε holds more of the activating NF-κB subunits c-Rel and p65. Therefore, when IκBε is knocked down, these activating NF-κB subunits transit to the nucleus where they bind to the HIV LTR, yielding an activating set of NF-κB subunits bound to the HIV LTR, leading to a large increase in HIV gene expression.

Limitations of this study include the fact that we used cell lines, U1, ACH-2, and J1.1. The U1 and ACH-2 cell lines have an altered Tat-TAR axis [75,76]. However, these alterations in Tat-TAR would not affect initial activation via NF-κB. If anything, the Tat-TAR alterations would lessen activation and help maintain latency, so evidence of effective activation via IκBε knockdown would be expected to be potent in cell lines without Tat-TAR alterations, as observed with the J1.1 cell line in this report and in our previous work [67]. Overall, the results were similar in all the cell lines tested, and were consistent with activation observed in our prior study [67].

Knocking down IκBα led to transit to the nucleus of mostly c-Rel, while knocking down IκBε led to the nuclear transit of larger amounts of both c-Rel and p65 (Figure 3A,B), with more p65 than c-Rel. Both p65 and c-Rel have TADs and so can form an activating heterodimer with, for example, p50 [74,77]. The larger amounts of c-Rel and p65 released for transit to the nucleus following IκBε knockdown offer a biologically plausible explanation for the enhanced activation activity of IκBε knockdown, particularly since an activating heterodimer with p50 can be formed with either c-Rel or p65, so releasing activating NF-κBs from a single IκBε can yield two activating heterodimers.

In some cell lines (ACH-2, J1.1) the efficiency of IκBε knockdown was slightly better than IκBα. In U1 cells, the knock down abilities of the IκBα and IκBε siRNAs were essentially equivalent (Figure 1), yet IκBε siRNA was a more efficient HIV activator in the U1 cells, as well as in the ACH-1 and J1.1 cell lines, arguing that specifically targeting IκBε as an HIV activating strategy could be potentially helpful. However, even if IκBε knockdown was less efficient than IκBα knockdown, the likely high therapeutic index of potential agents targeting IκBε, given the minimal phenotype observed in IκBε KO mice [66] as well as the slower resynthesis kinetics of IκBε [67], suggests that further efforts aimed at developing therapeutics targeting IκBε for HIV activation could be helpful.

While many HIV LARs for use in “shock and kill” strategies to attack and deplete the reservoir of long term latently infected cells have been studied, none have really been proven to be clinically safe and effective [1,2,3,4,5,6,16,18,19]. This unfortunate experience suggests that it might be helpful to pursue mechanistically novel latency activation technologies for use in “shock and kill.” Indeed, it may be necessary ultimately to combine multiple therapeutics targeting several different latency activation mechanisms (DNA methylation and HDAC inhibitors, P-TEFb releasing agents, histone methyl transferase inhibitors, as well as agents targeting the NFκB system) to effectively accomplish the “shock” of the “shock and kill” approach to HIV reservoir depletion [78]. Given the toxicity exhibited by more traditional agents targeting the NFκB system, such as phorbol esters and their derivatives, and inflammatory cytokines, a more gentle and specific approach narrowly targeting IκBε alone could prove useful for an NFκB component of “shock” HIV activators.

The notable HIV expression activation observed when IκBε is specifically knocked down, suggests that efforts aimed at developing methods to activate HIV expression for use in “shock and kill” therapies through specific targeting of IκBε or IκBε-NF-κB subunit interactions, as opposed to developing general approaches to activate HIV non-specifically through NF-κB pathways, might offer a new and potentially effective path to attacking the latent HIV reservoir.
